# Status of Quality Control for Laboratory Tests of Medical Institutions in Korea: Analysis of 10 Years of Data on External Quality Assessment Participation

**DOI:** 10.3390/healthcare8020075

**Published:** 2020-03-27

**Authors:** Hyeongsu Kim, Sollip Kim, Yeo-Min Yun, Tae-Hyun Um, Jeonghyun Chang, Kun Sei Lee, Sail Chun, Kyu-Dong Cho, Tae-Hwa Han

**Affiliations:** 1 Department of Preventive Medicine, School of Medicine, Konkuk University, Seoul 05030, Korea; mubul@kku.ac.kr (H.K.); kunsei.lee@kku.ac.kr (K.S.L.); 2 Department of Laboratory Medicine, Inje University, Ilsan Paik Hospital, Goyang 10380, Korea; uthmd@hanmail.net (T.-H.U.); azacsss@naver.com (J.C.); 3 Department of Laboratory Medicine, School of Medicine, Konkuk University, Seoul 05030, Korea; 4 Department of Laboratory Medicine, University of Ulsan College of Medicine and Asan Medical Center, Seoul 05505, Korea; sailchun@amc.seoul.kr; 5 Big Data Department, National Health Insurance Service, Seoul 26464, Korea; kdcho@nhis.or.kr; 6 Health IT Center, College of Medicine, Yonsei University, Seoul 03722, Korea; taehwa.han@gmail.com

**Keywords:** external quality assessment, mandatory, participation, quality of laboratory testing

## Abstract

External quality assessment (EQA) is a commonly used tool to track the performance of laboratory tests. In Korea, EQA participation is not mandatory, and even basic data about EQA participation are not available. We used data of a 10-year period extracted from two databases (2009–2018): (1) the database of the National Health Insurance Service to calculate the number of medical institutions that claimed health insurance benefits, and (2) the database of the Korean Association of External Quality Assessment Service to calculate the number of medical institutions participating in EQA. The proportion of institutions that made claims for the performance of laboratory testing throughout the 10 years were 73.6%–76.0% for clinics, 91.9%–97.5% for long-term care hospitals, 97.9%–99.5% for small to medium hospitals, 99.6%–100% for general hospitals, and 100% for tertiary hospitals. The mean EQA participation rate of institutions that performed laboratory testing for the 10 years was 1.9% for clinics, 3.1% for long-term care hospitals, 27.7% for small to medium hospitals, 96.6% for general hospitals, and 100% for tertiary hospitals. The mean EQA participation of clinics, long-term care hospitals, and small to medium hospitals are increasing but is still not sufficient. Regulatory approaches are needed to increase participation rates. This result would be used for health policymaking on the quality improvement of laboratory tests.

## 1. Introduction

In modern medicine, clinical laboratory data comprise a very large portion of patient care. More than half of a doctor’s decisions are thought to be influenced by laboratory data [1]. Nearly 94% of electronic medical record requests in one large medical center that monitors information flow were for laboratory testing [2]. Poor quality laboratory tests have significant financial, health, and social impacts, and quality improvement is very beneficial for the future [3]. 

It is reasonable that quality control efforts are relative to test complexity. In the US, laboratory tests are categorized as waived or moderate- or high-complexity tests under the Clinical Laboratory Improvement Amendments (CLIA) [4]. Waived tests are defined as tests with low risk for an incorrect result; so simple that less skilled persons can do them. In contrast, moderate- or high-complexity tests should be performed in the laboratories meeting CLIA quality standards, including external quality assessment (EQA) participation [5]. In Korea, there is not yet such a regulatory classification system, although relatively simple tests are notified in insurance benefits with the names of "handy tests" or “point-of-care tests”. “Handy tests” or “point-of-care tests” can be regarded as equivalent to the waived tests of the CLIA.

EQA is widely used as a tool to monitor laboratory quality [6]. EQA assesses the analytical performance of a laboratory relative to its peers (laboratories using the same method or instrument), reference standards, and/or reference laboratories [7]. When a participating laboratory submits its results for EQA samples, the EQA program evaluates and reports the performance of the laboratory based on statistical methods. The laboratories review their EQA evaluation reports and take action if necessary, which helps to improve the quality of the laboratory. As such, EQA is an essential tool for laboratory quality improvement, and most of the laboratory accreditation programs require EQA participation [7]. EQA helps laboratories recognize and resolve procedural weaknesses, and instills trust in the staff [8]. In 53% of Mediterranean countries (including France, Italy, and Turkey), the participation in EQA is mandatory by law, 29% of countries have guidelines for the scientific society, and, in 6% of countries (e.g., Greece), this is required by social security organizations for reimbursement of test costs [9]. In Germany, EQA participation is mandatory for designated test items [10]. In the United States, laboratories conducting moderate- or high-complexity tests are required to participate in EQA programs approved by the Center for Medicare and Medicaid Services under the Clinical Laboratory Improvement Amendment Act, which applies to all laboratories handling human samples [11]. In Korea, however, it is not mandatory and the very basic data such as EQA participation rates of medical institutions are not available.

The Korean Association of External Quality Assessment Service (KEQAS) was launched in 1976 with the aim of improving the reliability of laboratory tests directly related to public health. KEQAS is certified as an EQA provider according to ISO/IEC 17043 in 2015 and is the nation’s authorized EQA institute for the standardization and quality management of laboratory tests of medical institutions in Korea. The number of KEQAS participants has increased every year and reached 1818 as of October 2019. KEQAS provides 69 EQA programs in 2020, which cover all disciplines of laboratory medicine.

In this study, we aim to evaluate the EQA participation status according to the types of medical institutions in Korea. This would be useful data for quality management and improvement for laboratory tests.

## 2. Materials and Methods 

### 2.1. Number of Medical Institutions Performing Laboratory Tests

#### 2.1.1. Types of Medical Institutions

Medical institutions are classified into five types: clinics, long-term care hospitals, “hospitals”, general hospitals, and tertiary hospitals, according to the Korean Medical Service Act [12]. Clinics are medical institutions where doctors conduct their own medical practice, mainly for outpatients. Long-term care hospitals are hospitals for practicing medicine for patients who need long-term hospitalization. “Hospitals” have more than 30 beds. Since the term “hospital” is also a generic noun that includes many kinds of hospitals, we decided to equate the "hospital" of the Korean Medical Service Act to a “small to medium hospital” to prevent confusion in this paper. General hospitals must have more than 100 beds with seven or more predefined medical departments (in case of general hospitals with 100–300 beds), or nine or more medical departments (in case of general hospitals with >300 beds). Tertiary hospitals must have at least 20 medical departments set by Health and Welfare Ministry decree. 

#### 2.1.2. Classification of Laboratory Tests by Complexity

Laboratory tests can be classified as low-complexity tests and moderate- or high-complexity tests. Because there is no formal classification of complexity of tests in Korea, we regarded the tests with “handy” or “point-of-care” in the insurance benefits lists as low-complexity tests (which are similar to waived tests in CLIA), while the other tests as moderate- or high-complexity tests. The tests regarded as low-complexity are listed in Table S1.

#### 2.1.3. Number of Medical Institutions According to Laboratory Complexity

The research database of the National Health Insurance Service (NHIS) of Korea during the 10 years (2009–2018) was analyzed [13]. The number of medical institutions with any health insurance claims was regarded as the number of total medical institutions in Korea because all medical institutions must be registered for health insurance by law. We calculated the number of medical institutions with claims of any laboratory tests (LAB). Then we grouped medical institutions according to laboratory complexity and calculated their numbers: medical institutions with claims of moderate- or high-complexity laboratory tests (group LAB1), and medical institutions with claims of low-complexity tests only (group LAB2). LAB is subdivided into group LAB1 and group LAB2 (Table 1).

### 2.2. Medical Institutions with EQA Participation

Using the KEQAS database, we calculated the number of medical institutions with EQA participation (group EQA) according to the type of medical institutions for the 10 years (2009–2018). The EQA consists of dozens of programs, and each program has several test items. Medical institutions with enrollment in one or more EQA programs were regarded as EQA participation. 

### 2.3. EQA Participation Rate According to Medical Institution Types 

The EQA participation rate was defined as the percentage of medical institutions with EQA participation among medical institutions with laboratory testing (EQA participation rate = group EQA/group LAB × 100). 

### 2.4. Ethics Statement

The study was approved by the Institutional Review Board of Konkuk University, Seoul, in Korea (approval number: 7001355-202001-E-106).

## 3. Results

### 3.1. Number of Medical Institutions Performing Laboratory Tests

The number of medical institutions increased over the 10-year period except for tertiary hospitals. The proportion of institutions that claimed to perform laboratory testing (LAB) for the 10-year period were 73.6%–76.0% for clinics, 91.9%–97.5% for long-term care hospitals, 97.9%–99.5% for small to medium hospitals, 99.6%–100% for general hospitals, and 100% for tertiary hospitals (Table 1). 

The proportion of institutions claiming to perform moderate- or high-complexity laboratory testing (group LAB1) was 62.7%–66.2% for clinics, 75%–93.4% for long-term care hospitals, 96.7%–97.9% for small to medium hospitals, 99.6%–100% for general hospitals, and 100% for tertiary hospitals (Table 1).

The proportion of institutions claiming to perform laboratory testing low-complexity tests only (group LAB2) was 1.1%–1.8% for clinics, 0.1%–16.9% for long-term care hospitals, 0.0%–2.8% for small to medium hospitals, 0.0% for general hospitals, and 0.0% for tertiary hospitals (Table 1).

### 3.2. EQA Participation Rate According to Medical Institution Types

**Figure 1:**
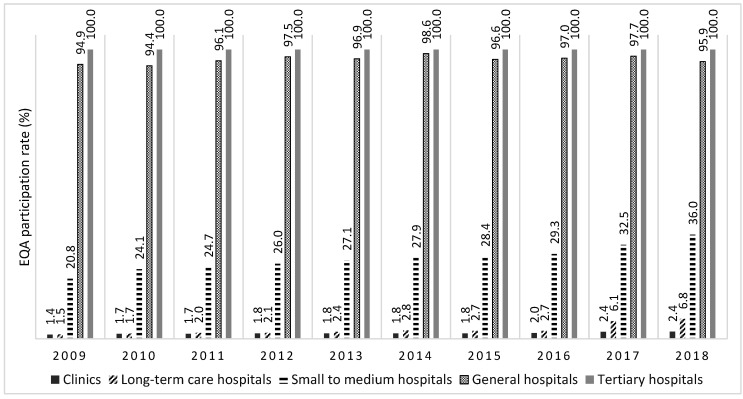
EQA participation rate of medical institutions with claims of laboratory testing for the 10 years (2009–2018).

The numbers and percentages of medical institutions participating in the EQA (group EQA) increased from 286 (1.4%) in 2009 to 556 (2.4%) in 2018 for clinics, 12 (1.5%) to 101 (6.8%) for long-term care hospitals, 278 (20.8%) to 572 (36.0%) for small to medium hospitals, and 261 (94.9%) to 303 (95.9%) for general hospitals. Those of the tertiary hospitals were from 44 (100%) to 42 (100%) (Figure 1).

The mean EQA participation rate for the 10 years was 1.9% for clinics, 3.1% for long-term care hospitals, 27.7% for small to medium hospitals, 96.6% for general hospitals, and 100% for tertiary hospitals. The EQA participation rates for clinics, long-term care hospitals, and small to medium hospitals have increased. 

## 4. Discussion

Most long-term care hospitals, small to medium hospitals, general hospitals, and tertiary hospitals, and even many clinics in Korea, perform laboratory testing. The mean EQA participation rate of tertiary hospitals and general hospitals in 2009–2018 was 100% and 96.6% for the 10 years, respectively. The EQA participation rate among clinics, long-term care hospitals, and small to medium hospitals increased in 2009–2018, but it was not sufficiently high. 

EQA participation is burdensome because it needs time, effort, and money. Clinicians also often do not realize the importance of quality control of laboratory testing. This may be why EQA participation rates in clinics, long-term care hospitals, and small to medium hospitals are low. However, despite this hassle, parts of these medical institutions participated in EQA, and participation rates are slowly increasing. Although, the root cause is unknown, since this study is based on secondary data analysis, presumably, the increase of EQA participation rate in clinics, long-term care hospitals, and small to medium hospitals may be related to an increase in institutions conduct NHIS-supported public health checkups that require EQA participation by the governmental evaluation [14]. The number of medical institutions (excluding dental clinics, dental hospitals, and public health centers) performing NHIS-supported public health checkups increased from 6287 in 2009 to 10,143 in 2018 (unpublished data, Table S2). 

On the other hand, the mean EQA participation rate of tertiary hospitals and general hospitals was high. Hospitals with clinical pathologists (mainly general hospitals or tertiary hospitals) have always been interested in improving the quality of laboratory testing, and have participated for decades in the laboratory accreditation program that requires EQA participation. In addition, these have been subjected to government evaluation for medical institutions since 2004, which also requires EQA participation [15]. This may be why EQA participation rates in general hospitals and tertiary hospitals are consistently high. External assessments and legal enforcement of regulations are likely to lead to an increase in EQA participation. Compensation for the cost incurred in enforced EQA participation should be adequately covered by the NHIS. This is appropriate, given the time, effort, and money required for EQA participation.

We observed that although more than 90% of long-term care hospitals and more than 95% of small to medium hospitals performed moderate- or high-complexity tests, the EQA participation rate of long-term care hospitals was less than 10% and those of small to medium hospitals was less than 30%. This suggests that a significant number of long-term care hospitals and small to medium hospitals that perform moderate- or high-complexity tests do not participate in EQA programs. Moderate- or high-complexity tests require more stringent quality control because of the higher risk to patients from erroneous results. Test complexity should be considered when applying regulations to improve laboratory testing quality.

One of the study limitations is that this study only examines participation in EQA. The total quality change in each institution could, therefore, not be determined. Although EQA is widely used for monitoring the performance of clinical laboratories [6], EQA only measures performance quality at specific time points, which does not reflect the overall quality of laboratory performance. Participation in laboratory accreditation programs could be a good alternative for monitoring and improving the overall quality of laboratory testing. Jang et al. [16] reported that institutions with laboratory accreditation had significantly better EQA results over a 4-year study period for all general chemistry tests (*p* < 0.0001) compared to institutions without accreditation. They highlighted the importance of laboratory accreditation programs in improving laboratory testing. For better quality testing, laboratories should perform various quality improvement activities, including laboratory accreditation as well as EQA participation. In addition, regulations are needed to ensure that medical institutions that carry out laboratory tests are established only if proper facilities and personnel sufficient quality control are available.

Another limitation arose from a secondary data analysis of this study. That is, we were unable to specify the exact reason for the low participation rate among clinics, long-term care hospitals, and small to medium hospitals, and we were not able to analyze performance levels according to the type of facility. Further research is required to solve these questions with better data. However, this study might be good evidence for further research.

## 5. Conclusions

This is the first study to analyze EQA participation rates of medical institutions by type in Korea. The mean EQA participation rates of clinics, long-term care hospitals, and small to medium hospitals are increasing but remains unsatisfactory. Appropriate regulations are needed to increase EQA participation rates, especially of medical institutions that perform moderate- or high-complexity tests. Although our study findings are preliminary, they can help guide health policies to improve the quality of clinical laboratories.

## Figures and Tables

**Table 1:** The number of medical institutions according to the laboratory complexity (2009–2018).

Type	Group	2009	2010	2011	2012	2013	2014	2015	2016	2017	2018
Clinics	Total (%)	26,957 (100.0)	27,387 (100.0)	27,731 (100.0)	27,832 (100.0)	28,067 (100.0)	28,383 (100.0)	28,997 (100.0)	29,789 (100.0)	30,399 (100.0)	30,929 (100.0)
	LAB (%)	20,484 (76.0)	20,664 (75.5)	20,874 (75.3)	20,797 (74.7)	20,881 (74.4)	20,998 (74.0)	21,429 (73.9)	21,922 (73.6)	22,469 (73.9)	22,885 (74.0)
	LAB1 (%)	17,852 (66.2)	17,918 (65.4)	18,078 (65.2)	17,907 (64.3)	17,963 (64.0)	18,077 (63.7)	18,307 (63.1)	18,683 (62.7)	19,276 (63.4)	19,787 (64.0)
	LAB2 (%)	2632 (9.8)	2746 (10.0)	2796 (10.1)	2890 (10.4)	2918 (10.4)	2921 (10.3)	3122 (10.8)	3239 (10.9)	3193 (10.5)	3098 (10.0)
Long-term care hospitals	Total (%)	862 (100.0)	999 (100.0)	1117 (100.0)	1241 (100.0)	1360 (100.0)	1443 (100.0)	1482 (100.0)	1515 (100.0)	1541 (100.0)	1578 (100.0)
	LAB (%)	803 (93.2)	918 (91.9)	1031 (92.3)	1157 (93.2)	1280 (94.1)	1372 (95.1)	1419 (95.7)	1470 (97.0)	1503 (97.5)	1475 (93.5)
	LAB1 (%)	663 (76.9)	749 (75.0)	855 (76.5)	958 (77.2)	1091 (80.2)	1209 (83.8)	1317 (88.9)	1384 (91.4)	1428 (92.7)	1474 (93.4)
	LAB2 (%)	140 (16.2)	169 (16.9)	176 (15.8)	199 (16.0)	189 (13.9)	163 (11.3)	102 (6.9)	86 (5.7)	75 (4.9)	1 (0.1)
Small to medium hospitals	Total (%)	1353 (100.0)	1434 (100.0)	1499 (100.0)	1552 (100.0)	1564 (100.0)	1566 (100.0)	1580 (100.0)	1582 (100.0)	1616 (100.0)	1623 (100.0)
	LAB (%)	1339 (99.0)	1425 (99.4)	1491 (99.5)	1545 (99.5)	1554 (99.4)	1555 (99.3)	1566 (99.1)	1568 (99.1)	1608 (99.5)	1589 (97.9)
	LAB1 (%)	1315 (97.2)	1400 (97.6)	1449 (96.7)	1502 (96.8)	1514 (96.8)	1516 (96.8)	1532 (97.0)	1538 (97.2)	1568 (97.0)	1589 (97.9)
	LAB2 (%)	24 (1.8)	25 (1.7)	42 (2.8)	43 (2.8)	40 (2.6)	39 (2.5)	34 (2.2)	30 (1.9)	40 (2.5)	0 (0.0)
General hospitals	Total (%)	276 (100.0)	286 (100.0)	285 (100.0)	284 (100.0)	286 (100.0)	290 (100.0)	297 (100.0)	302 (100.0)	304 (100.0)	316 (100.0)
	LAB (%)	275 (99.6)	285 (99.7)	285 (100.0)	284 (100.0)	286 (100.0)	290 (100.0)	297 (100.0)	302 (100.0)	304 (100.0)	316 (100.0)
	LAB1 (%)	275 (99.6)	285 (99.7)	285 (100.0)	284 (100.0)	286 (100.0)	290 (100.0)	297 (100.0)	302 (100.0)	304 (100.0)	316 (100.0)
	LAB2 (%)	0 (0.0)	0 (0.0)	0 (0.0)	0 (0.0)	0 (0.0)	0 (0.0)	0 (0.0)	0 (0.0)	0 (0.0)	0 (0.0)
Tertiary hospitals	Total (%)	44 (100.0)	44 (100.0)	44 (100.0)	44 (100.0)	43 (100.0)	43 (100.0)	43 (100.0)	43 (100.0)	43 (100.0)	42 (100.0)
	LAB (%)	44 (100.0)	44 (100.0)	44 (100.0)	44 (100.0)	43 (100.0)	43 (100.0)	43 (100.0)	43 (100.0)	43 (100.0)	42 (100.0)
	LAB1 (%)	44 (100.0)	44 (100.0)	44 (100.0)	44 (100.0)	43 (100.0)	43 (100.0)	43 (100.0)	43 (100.0)	43 (100.0)	42 (100.0)
	LAB2 (%)	0 (0.0)	0 (0.0)	0 (0.0)	0 (0.0)	0 (0.0)	0 (0.0)	0 (0.0)	0 (0.0)	0 (0.0)	0 (0.0)

Total, Number of medical institutions with any health insurance claims; LAB, Number of medical institutions with claims of health insurance benefit with any laboratory test; LAB consists of LAB1 and LAB2; LAB1, Number of medical institutions with claims of health insurance benefit with moderate- or high-complexity laboratory tests; LAB2, Number of medical institutions with claims of health insurance benefit for low-complexity tests only.

## Supplementary Materials

The following are available online at https://www.mdpi.com/2227-9032/8/2/75/s1, Table S1: List of low-complexity tests, Table S2: The number of medical institutions performing NHIS-supported public health checkups (excluding dental clinics, dental hospitals, and public health centers). Data were from the Korean Statistical Information Service (KOSIS, http://kosis.kr/).
