# Supplementary material for: Status of Quality Control for Laboratory Tests of Medical Institutions in Korea: Analysis of 10 Years of Data on External Quality Assessment Participation
Source: Healthcare (Basel). 2020 Mar 27;8(2):75. doi: 10.3390/healthcare8020075 (PMC7349217; doi:10.3390/healthcare8020075)
Supplement: Supplementary file 1 [file healthcare-08-00075-s001.pdf]

healthcare

MDPI

Supplemental Table S1. List of low-complexity tests.

Test	Current claim code (after 2018.1)	Previous claim code (until 2017.12)
C-Reactive Protein, Chemical reaction-instrument reading-handy	D0114*	
MMP-9 [General immunoassay]-handy	D0120*	B0491 *
Amniotic fluid MMP-8 qualitative [POCT]	BZ011	
Platelet drug responsiveness (aspirin) [POCT]	BZ071	
Platelet drug responsiveness (P2Y12)) [POCT]	BZ072	
Fecal Calprotectin, General immunoassay (1) qualitative-handy	D0131*	B0751 *
Fecal Calprotectin, General immunoassay (1) quantitative-handy	D0132*	B0753 *
Prothrombin Time -handy	D1008*	B1541
Activated clotting time-handy	D1014*	
Direct Bilirubin, Chemical reaction-instrument reading-handy	D1821*	
Total Bilirubin, Chemical reaction-instrument reading-handy	D1831*	
Total Protein, Chemical reaction-instrument reading-handy	D1841*	
ALT Chemical reaction-instrument reading-handy	D1851*	
AST Chemical reaction-instrument reading-handy	D1861*	
Alkaline Phosphatase, Chemical reaction-instrument reading-handy	D1871*	
Albumin, Chemical reaction-instrument reading-handy	D1881*	
γ-GTP, Chemical reaction-instrument reading-handy	D1891*	
Creatinine, Chemical reaction-instrument reading-handy	D2281*	
Uric Acid, Chemical reaction-instrument reading-handy	D2311*	
Neutrophil Gelatinase-Associated Lipocalin (NGAL), Precise immunoassay-handy	D2341*	CZ247
Enzyme, Chemical reaction-instrument reading-handy (01) CPK	D2511*(01)	
Lipid [Chemical reaction-instrument reading] 마. Triglyceride -handy	D2265*	
Total Cholesterol, (2) Chemical reaction-instrument reading-handy	D2616*	
HDL Cholesterol, (2) Chemical reaction-instrument reading-handy	D2618*	
Ketone Body, Chemical reaction-instrument reading-handy	D3012	C3861
Glucose [Chemical reaction-instrument reading] quantitative, glucometer	D3021	C3710
Hemoglobin A1c, General immunoassay-handy	D3062	
Gonadotropin, General immunoassay (qualitative) - handy (01) Luteinizing Hormone	D3701(01)	
Digestive Tract Enzyme, Chemical reaction-instrument reading-handy (01) Amylase (Total)	D3801*(01)	
Troponin, General immunoassay (qualitative) - handy (01) Troponin I	D4021(01)	
Troponin, General immunoassay (qualitative) - handy (02) Troponin T	D4021(02)	
Troponin, Precise immunoassay (quantitative) - handy (01) Troponin I *	D4022*(01)	CY279
Troponin, Precise immunoassay (quantitative) - handy (02) Troponin T *	D4022*(02)	

Cardiac Marker, Precise immunoassay-handy (01) Pro-Brain Natriuretic Peptide	D4061(01)	
Cardiac Marker, Precise immunoassay-handy (02) Brain Natriuretic Peptide	D4061(02)	
Bladder Tumor Antigen, General immunoassay (qualitative) -handy (01) NMP22	D2221(01)	
Premature Rupture of Membrane Test, General immunoassay-handy (01) Phosphorylated Insulin-like Growth Factor Binding Protein-1 (phIGFBP-1)	D5711*(01)	B0114 *
Premature Rupture of Membrane Test, General immunoassay-handy (02) Insulin-Like Growth Factor Binding Protein-1	D5711*(02)	BX014
Premature Rupture of Membrane Test, General immunoassay-handy (03) Placental Alpha-1 Microglobulin Protein	D5711*(03)	B0562
Premature Rupture of Membrane Test, General immunoassay-handy (04) qualitative Fetal Fibronectin	D5711*(04)	BX013
General immunoassay, Urine bacterial antigen-handy Urinary Antigen (01) Legionella	D1584*(01)	C5310
General immunoassay, Urine bacterial antigen -handy Urinary Antigen (02) Streptococcus Pneumoniae *	D1584*(02)	CZ398 *
Clostridium difficile toxin A·B, Glutamate dehydrogenase [Precise immunoassay]-handy	D5904*	B4070 *
Malaria Antigen (Plasmodium lactate dehydrogenase) [Rapid test]	CZ397*	CZ397 *
HCV Rapid Antibody Test	CZ492*	CZ492 *
Norovirus Antigen Test [Rapid Test]	CZ493*	CZ493 *
Hemoglobin A1c, Subfraction (03) chromatography-handy	D3064(03)	
Influenzavirus A·B antigen [POCT]	CZ394	CZ394
HIV antibody [POCT]	CZ396*	CZ396
Urine Pregnancy Test, General immunoassay (qualitative)	D5701	B0260
Urine, Chemical reaction-visual reading †	D2241	
Urinalysis [Chemical reaction-visual reading /Chemical reaction-instrument reading] Routine Urinalysis, Up to 4	D2251	B0010
Urinalysis [Chemical reaction-visual reading /Chemical reaction-instrument reading] Routine Urinalysis, Up to 7	D2252	B0020
Urinalysis [Chemical reaction-visual reading /Chemical reaction-instrument reading] Routine Urinalysis, Up to 10	D2253	B0030

Abbreviation: POCT, point-of-care testing.

Supplemental Table S2. The number of medical institutions performing NHIS-supported public health checkups (excluding dental clinics, dental hospitals and public health center). Data were from the Korean Statistical Information Service (KOSIS, http://kosis.kr/).

Year	2009	2010	2011	2012	2013	2014	2015	2016	2017	2018
Clinics	5,040	6,113	6,620	7,026	7,315	7,573	7,872	8,240	8,448	8,658
Long-term care hospitals and small to medium hospitals	938	969	1,018	1,072	1,071	1,100	1,108	1,150	1,162	1,142
General hospitals and tertiary hospitals	309	310	312	315	316	323	329	333	336	343
Total	6,287	7,392	7,950	8,413	8,702	8,996	9,309	9,723	9,946	10,143
